# The Role of Endoscopic Ultrasound in the Interventional Management of Mediastinal Collections: A Narrative Review

**DOI:** 10.7759/cureus.27803

**Published:** 2022-08-09

**Authors:** Julio G Velasquez-Rodriguez, Sandra Maisterra, Ricard Ramos, Ignacio Escobar, Joan B Gornals

**Affiliations:** 1 Digestive Diseases/Endoscopy, Hospital Universitari de Bellvitge/Universitat de Barcelona, Barcelona, ESP; 2 Clinical Research, Bellvitge Biomedical Research Institute (IDIBELL), Barcelona, ESP; 3 Department of Clinical Sciences, School of Medicine and Health Sciences, University of Barcelona, Barcelona, ESP; 4 Thoracic Surgery, Hospital Universitari de Bellvitge/Universitat de Barcelona, Barcelona, ESP; 5 Faculty of Health Sciences, Universitat Oberta de Catalunya (UOC), Barcelona, ESP

**Keywords:** mediastinal pseudocyst, interventional endoscopy, therapeutics, transmural drainage, endoscopic ultrasound, mediastinal cyst, mediastinal collection

## Abstract

The numerous causes underlying mediastinal lesions require different diagnostic and therapeutic approaches, including conservative, minimally invasive, and surgical interventions. Solid lesions of a malignant nature, mostly located in the anterior mediastinum, are properly treated with surgical resection either with or without adjuvant schemes. In contrast, a surveillance program is usually recommended with solid benign tumors, depending on their size and related symptomatology. In the management of mediastinal collections, when a drainage intervention is required (suspicion of infection and symptomatology), a minimally invasive nonsurgical procedure or thoracic surgery is considered. The minimally invasive nonsurgical procedures that can be available are percutaneous radiology-guided imaging (abdominal ultrasound (US) or computed tomography (CT) scan), complete single-aspiration guided by endoscopic ultrasound (EUS) or endobronchial ultrasound (EBUS), and transmural drainage guided by EUS. Surgical debridement is feasible to treat collections, but as this entails considerable risk of postoperative complications, it is chosen only when other minimally invasive therapies are not possible. The published literature related to the interventional endoscopic approach to mediastinal lesions is scarce. Nevertheless, reports in this field reveal that interventional EUS may have a role in both the diagnosis of and therapeutic approach to mediastinal lesions, mainly in the management of mediastinal collections.

## Introduction and background

Mediastinal lesions include a broad spectrum of pathologies. According to their nature, they may complicate locally with a mass effect on adjacent organs [1]. These lesions are divided into primary (e.g., solid or cystic lesions) and secondary (e.g., infectious, inflammatory, or hemorrhagic lesions) [2]. Multiple treatment options for mediastinal lesions exist, such as conservative management, minimally invasive procedures, and surgical interventions. Endoscopic ultrasound (EUS) is in most cases limited to posterior mediastinum-located lesions, offering an interventional ability to obtain diagnostic samples of mediastinal lesions.

The management of mediastinal collections that require drainage includes a surgical approach or a minimally invasive nonsurgical procedure such as percutaneously guided imaging or endoscopically by EUS.

The main objective of this review is to highlight the management of mediastinal collections (mainly posterior or middle) with simple (single aspiration) (safer but higher relapse rate) or transmural drainage (stenting) (high efficiency but more aggressive) guided by EUS.

## Review

Methodology

We conducted a traditional narrative review (non-systematic and not including all available evidence) for obtaining a broad perspective on this topic. The main purpose was to identify peer-reviewed, published studies evaluating the role of interventional EUS in the management of mediastinal collections.

The literature cited in this narrative review was published from 1973 to 2022 and was searched on PubMed and Google Scholar. The following keywords were used: “mediastinal collection,” “mediastinal cyst,” “mediastinal pseudocyst,” “endoscopic ultrasound,” “fine needle aspiration,” and “transmural drainage.” The reviewers selected these articles by reviewing their titles and abstracts. Eligible studies considered relevant for drafting this narrative review included those that evaluated mediastinal collections or mediastinal pseudocyst treated by endoscopic ultrasound guidance (single aspiration, drainage, or pancreatic stent in the case of mediastinal pseudocyst). Additionally, the reference lists of the included studies were hand-searched.

Classification of mediastinal lesions

The mediastinum is a division of the thoracic cavity that has two main compartments: superior and inferior. The inferior is the larger one and so is further subdivided into three compartments: anterior, middle, and posterior [3].

**Figure 1:**
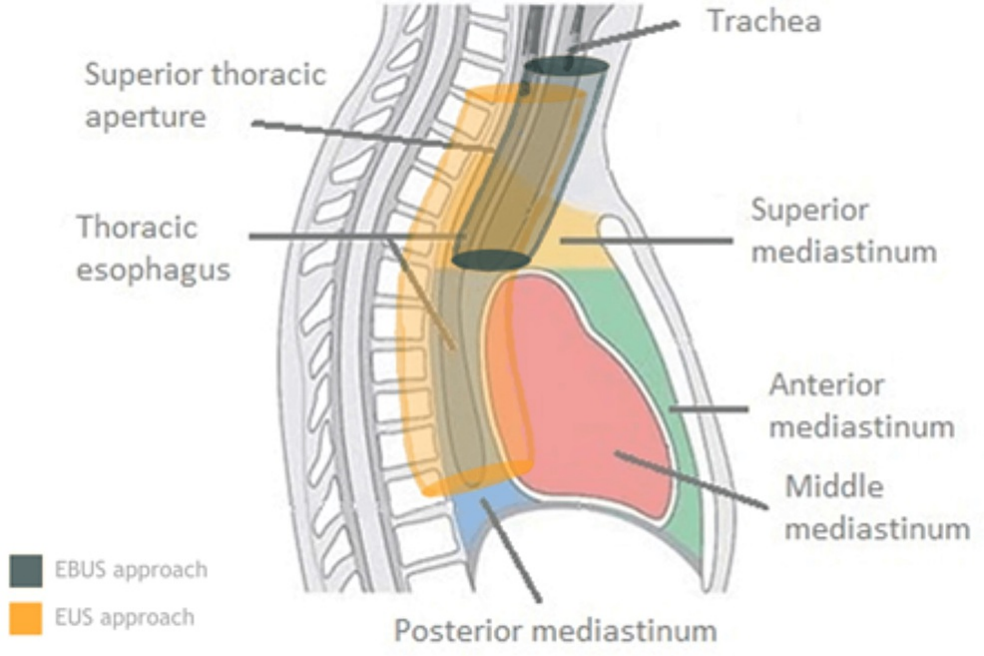
Compartments of the mediastinum The two main compartments are the superior and inferior mediastinum (including anterior, middle, and posterior). The location of the trachea and esophagus is essential for understanding the role and limitations of EBUS and EUS, respectively.

This division accurately accounts for localizing and characterizing mediastinal lesions. Firstly, the mediastinum is divided into superior and inferior compartments by a plane that runs from the sternal angle to the T4-T5 vertebrae. The superior mediastinum is limited by the root of the neck at the top of the first ribs and by the inferior mediastinum below the described transverse thoracic plane. Secondly, the inferior mediastinum is divided into the anterior, middle, and posterior mediastinum, as shown in Figure 1.

Table 1 presents the main structural contents of each mediastinal compartment.

**Table 1:** Mediastinum contents by compartment

	Superior mediastinum	Inferior mediastinum		
		Anterior	Middle	Posterior
Organs	Thymus, trachea, and esophagus	Thymus	Heart pericardium	Esophagus, thoracic spine, and paravertebral soft tissues
Arteries	Aortic arch, brachiocephalic trunk, and left common carotid	Smaller vessels and left brachiocephalic trunk	Ascending aorta, pulmonary trunk, branches, and pericardiacophrenic	Thoracic aorta and branches
Veins and lymph vessels	Superior vena cava, brachiocephalic trunk, and thoracic duct	Smaller vessels, lymphatics, and lymph nodes	Superior vena cava, azygos, pulmonary, and pericardiacophrenic	Azygos, hemiazygos, and thoracic duct
Nerves	Vagus, phrenic, and left recurrent laryngeal	None	Phrenic	Vagus

Mediastinal lesions are clustered according to their tumor or cystic formation as malignant or benign course and primary or secondary origin. Following this classification, we may carry out differential diagnoses and identify potential treatment approaches.

Table 2, Table 3, and Table 4 include most of the mediastinal lesions, arranged according to their locations [1,4-28].

Lesions of the anterior mediastinum are solid tumors; thus, their treatment is usually based on surgical intervention. Some exceptions, where first-line treatment includes chemotherapy, exist, such as lymphoma (Table 2) [1,29-38]. In the middle mediastinum, most lesions are primary benign cysts such as bronchogenic cysts (Table 3) [32,33]. In the posterior mediastinum, most lesions are benign secondary collections, mainly liquid-containing lesions, with pseudocysts and abscesses being the most prevalent. More details concerning this group are presented in Table 4.

**Table 2:** Mediastinal lesions according to topographic location (prevascular (or anterior) mediastinum) CT: chemotherapy; CT-FNA: computed tomography-guided fine needle aspiration; CT scan: computed tomography scan; EBUS-TBNA: endobronchial ultrasound-guided transbronchial needle aspiration; HD: Hodgkin disease; MEN1: multiple endocrine neoplasia type 1; MRI: magnetic resonance image; RT: radiotherapy; SurgT: surgical treatment; SVCS: superior vena cava syndrome; US-FNA: ultrasound-guided fine needle aspiration

Differential diagnosis	Features	Diagnosis	Approach
Thymus epithelial tumors			
Thymoma	Solid tumor, malignant, associated with myasthenia gravis	Contrasted CT scan, CT-FNA, US-FNA (staging)	SurgT ± CT ± RT
Thymic carcinoma	Malignant, symptomatic, associated with pleural effusion and pericardial cyst [1]	Contrasted CT scan, CT-FNA, US-FNA (staging)	SurgT, unresectable: CT/RT
Thymic carcinoid	Malignant, associated with Cushing syndrome and MEN1 [29],metastasis frequently	Contrasted CT scan to rule out hemorrhage, necrosis	SurgT ± CT/RT
Fat content lesions			
Thymolipoma	Rare, benign	MRI better than CT scan in differing fat	SurgT
Lipoma	Rare, benign, incidental	Contrasted CT scan	SurgT if with symptoms
Thymus hyperplasia	Young patient, follicular type associated with myasthenia gravis [30]	MRI better than CT scan in ruling out neoplastic features [8]	SurgT if with symptoms
Cystic lesions			
Thymic cyst	Rare, unilocular/multilocular, associated with inflammation or neoplasm (HD)	Contrasted CT scan; if solid component, MRI	SurgT if with symptoms
Pericardial cyst	Benign, right cardiophrenic angle [31]	Contrasted CT scan: unilocular, non-enhancing	SurgT if with symptoms
Lymphangioma	Congenital, benign, 10% mediastinal, associated with chylothorax and hemangiomas	CT scan, lymphangiography [32]	SurgT if with symptoms, visceral mediastinum, EBUS-TBNA [33]
Lymphoma	Rare, 10% of lymphoma, Hodgkin disease	CT scan	CT, RT
Germ cell tumors			
Teratoma	Benign, asymptomatic	CT scan	SurgT if with symptoms
Seminoma [37,38]	Men, 20-40 years, until 10% SVCS [34]	Gallium CT scan [35]	RT, CT, SurgT

**Table 3:** Mediastinal lesions according to topographic location (visceral (or middle) mediastinum) CT scan: computed tomography scan; EBUS: endobronchial ultrasound; EBUS-TBNA: endobronchial ultrasound-guided transbronchial needle aspiration; EGD: esophagogastroduodenoscopy; EUS-FNA: endoscopic ultrasound-guided fine needle aspiration; SurgT: surgical treatment; VATS: video-assisted thoracoscopic surgery

Differential diagnosis	Features	Diagnosis	Approach
Cystic lesions			
Bronchogenic cyst	Cartilaginous content, near the carina	CT scan, EBUS, EUS (sample)	SurgT, single aspiration
Esophageal cyst	Esophageal wall	CT scan, EUS	SurgT, VATS
Pericardial cyst	Congenital, benign; right cardiophrenic angle		
Lymphangioma	Congenital, benign, 10% mediastinal, associated with chylothorax and hemangiomas	CT scan, lymphangiography [32]	SurgT if with symptoms, visceral mediastinum, EBUS-TBNA [33]
Esophageal lesions			
Esophageal cancer	Dysphagia	EGD, CT scan, EUS	SurgT (T1a: endoscopy)
Esophageal leiomyoma	Benign	EUS	Conservative, endoscopy, SurgT
Metastasis		CT scan, EUS-FNA	According to primary

**Table 4:** Mediastinal lesions according to topographic location (paravertebral (or posterior) mediastinum) CT: chemotherapy; CT-FNA: computed tomography-guided fine needle aspiration; CT scan: computed tomography scan; EUS-FNA: endoscopic ultrasound-guided fine needle aspiration; SurgT: surgical treatment; RT: radiotherapy; US-FNA: ultrasound-guided fine needle aspiration

Differential diagnosis	Features	Diagnosis	Approach
Neurogenic tumors			
Nerve sheath tumors	Benign	CT scan, MRI for intraspinal extension	SurgT ± CT, RT
Autonomic ganglionic tumors	Malignant, associated with neurofibromatosis, 5% sarcomatous degeneration	CT scan	
Cystic lesions			
Pancreatic pseudocyst	Acute or chronic pancreatitis history	CT scan to rule out necrosis, EUS-FNA	Conservative, SurgT, EUS drainage, transpapillary (via ERCP)
Mediastinal abscess	Esophageal surgery or injury history	CT scan to rule out associated empyema	Conservative, SurgT, EUS drainage

Mediastinal collections in the posterior mediastinum

Lesions located in the posterior mediastinum are most commonly punctured or treated by EUS. As previously commented, most of these lesions are abscesses and pancreatic fluid collections (pseudocyst or rarely walled-off pancreatic necrosis).

Mediastinal Abscess

A mediastinal abscess can be fatal, with mortality rates reaching 40%, especially when proper diagnosis and treatment are delayed [4]. Injury of adjacent organs such as esophageal perforation, sternal osteomyelitis, and extension of pulmonary, pleural, cervical, or retroperitoneal infection are the primary causes of associated morbimortality. Classically, antibiotic therapy in combination with surgical debridement, either by open surgery or video-assisted, is the treatment of choice [5]. Nevertheless, minimally invasive procedures, such as percutaneous drainage, have proven effective, and they carry lower complication rates [6].

Pancreatic Fluid Collections

Pancreatic fluid collections, mainly pseudocysts, are the second cause of secondary mediastinal lesions. They develop by encapsulation with an inflammatory tissue wall, which appears in the background of acute or chronic pancreatitis. Due to the location of the pancreas, the pancreatic fluid collection grows throughout the retroperitoneal region; however, it may be allocated in other cavities such as the mediastinum. The lesion reaches the mediastinum typically through the diaphragmatic hiatus and less commonly via the aorta or inferior cava hiatus vein. Most pancreatic fluid collections resolve spontaneously [7]. In accordance with lesion features such as size, related symptom severity, pancreatic ductal anatomy, and operator experience, the need for therapy will be discussed below.

Diagnosis of mediastinal lesions

Diagnostic and Interventional Imaging

It appears that a CT scan is equal to or slightly superior to magnetic resonance imaging (MRI) in the diagnosis of anterior mediastinal tumors, except for thymic cysts. Some studies analyzing and comparing the sensitivity of CT scan images to MRI in mediastinum lesion diagnosis reported only a slight superiority of CT scan, with an accuracy of 61% compared to 56%. They optimized the diagnosis, putting the two together and reaching 67% accuracy [8]. However, MRI benefits were the focus of the study of cystic lesions since it is capable of identifying malignancy signs, such as solid components, and areas of necrosis, and also explicitly distinguishing thymus hyperplasia from thymus neoplasia [8].

Transthoracic ultrasound (US) has proven its utility in the study of mediastinal lesions since it offers the advantage of sampling via US-guided fine needle aspiration (US-FNA). Nevertheless, the benefits are limited to the anterior mediastinum [9].

Interventional Diagnosis by EBUS

 Mediastinal lesion samples obtained by either EBUS-guided transbronchial needle aspiration (TBNA) or endoscopic ultrasound-guided tissue acquisition (EUS-TA) show an average sensitivity of 93%, a result that is notably valuable regarding lymphoma and other mediastinal neoplastic studies since diagnosis requires immunohistochemical evidence [10]. These are minimally invasive procedures that permit the avoidance of more aggressive interventions such as mediastinoscopy and thoracoscopy and thereby offer a lower complication rate [11]. In addition, it is important to consider anatomical references to better understand the range of intervention capacity for each procedure. Thus, EBUS-TBNA is performed with a bronchoscope using a transducer on the tip, which enables approaching the peribronchial or peritracheal areas to either sample tissue or drain aspiration. Furthermore, lesions located in the anterior mediastinum are still not accessible by EBUS-TBNA.

Interventional Diagnosis by EUS of Middle and Posterior Mediastinal Lesions

EUS-TA offers the power to acquire tissue from the middle and posterior mediastinum along the esophagus for cytohistological study with significant accuracy and safety [12,13,18,39-45]. A study that compared the usefulness of EBUS-TBNA versus EUS-FNA in the diagnosis of mediastinal lesions under suspicion of malignancy did not uncover differences between the two procedures, and by combining them, researchers achieved nearly 100% accuracy [12]. These results suggest the utility of combining the two procedures to inspect and sample within the different mediastinum regions.

In contrast, although they are both considered minimally invasive interventions compared to the classically used methods such as mediastinoscopy, there could still be complications. A national retrospective survey evaluated the rate of adverse events related to EBUS-TBNA and EUS-FNA. For a 12-year period, the study obtained an adverse event rate of 0.11% and 0.16% related to EBUS-TBNA and EUS-FNA, respectively, and found an overall mortality rate of 0.04% [13]. Most complications were infectious diseases related to tissue acquisition; bleeding was less frequent and better controlled during procedures. A technical advantage of EUS-TA also includes the capability of using a wider-diameter needle such as 19-gauge needles [14].

Treatment approaches for mediastinal collections

Conservative Management Strategy

Conservative treatment is considered the first step of therapy only if the patient is suitable for this approach. This involves the lesion type and its clinical impact. Mediastinal collections such as pancreatic pseudocyst can be handled by diverse approaches. Case reports have shown the efficacy of conservative treatment with octreotide, a somatostatin analog that inhibits pancreatic secretions and facilitates the closing of the pancreatic fistula, which forms the collection [15-17]. Nonetheless, the evidence for clinical recommendation of the treatment is lacking. On the other hand, mediastinal abscess shows a poor response to conservative treatment. Therefore, a better approach would be surgical drainage and then minimally interventional procedures.

Interventionist Treatment

Patients who are nonresponders to conservative treatment of lesions are considered for therapeutic intervention, aiming at debridement and/or drainage. In this setting and because of overall morbidity especially in nonsurgical candidates, minimally invasive techniques have been offered and have shown satisfactory results. These techniques include CT-guided percutaneous drainage, EUS- or EBUS-guided single needle aspiration, and/or EUS-guided transmural drainage. Decisions on strategy therapy should be made by a multidisciplinary committee. The use of C02 is highly recommended for EUS-guided transmural procedures to prevent air embolism.

CT-Guided Percutaneous Drainage

CT-guided percutaneous drainage represents an appropriate alternative to interventionist treatment. Since the first report in 1987 of a mediastinal abscess treated by CT-guided percutaneous drainage, several case reports and a 10-year series including 23 patients have been published [6,25-28]. Some 84% of abscesses were located in the posterior mediastinum, while the remainder were located in the anterior mediastinum. Technical success was achieved in 100% of procedures, while clinical success, which was defined as abscess resolution by a percutaneous approach without the need for surgical debridement, was achieved in 22 of 23 patients (96%) [6]. However, studies comparing this procedure to surgical debridement and endoscopic management are lacking.

EBUS-TBNA Drainage (Single Aspiration)

There are a few data concerning treatment using the EBUS-TBNA approach. Table 5 summarizes case reports of successful EBUS-TBNA treatment [36,46-53]. These reports suggest that EBUS-TBNA could be a safe alternative treatment compared to more aggressive strategies. The limitations of this approach include the use of narrow devices such as 22- or 25-gauge needles, which preclude performing drainage by single aspiration only. Furthermore, as previously mentioned, mediastinal access to the EBUS-TBNA is limited, so almost all reported cases listed involve lesions located in the paratracheal area within the middle mediastinum.

**Table 5:** Mediastinal lesions treated using EBUS-TBNA single aspiration * Disappearance of the lesion or enough reduction to exclude further procedures EBUS-TBNA: endobronchial ultrasound-guided transbronchial needle aspiration; NA: not applicable

Author, year	Number of patient(s)	Diagnosis	Size	Materials	Clinical success*	Stent removal	Adverse events	Follow-up
Nakajima et al., 2007 [48]	1	Mediastinal cyst	65 × 57 mm	Single EBUS-TBNA, 22 Ga	Yes	NA	No	1 year
Twehues et al., 2011 [49]	2	Bronchogenic cyst	40 × 56 mm	Single EBUS-TBNA, 22 Ga	Yes	NA	NA	16 months
		Bronchogenic cyst	43 × 57 mm	Single EBUS-TBNA, 22 Ga	No	NA	NA	NA
Choi et al., 2012 [36]	1	Paratracheal lymphangioma	137 mm (diameter)	Single EBUS-TBNA, 22 Ga	Yes	NA	No	12 months
Alraiyes et al., 2015 [50]	1	Bronchogenic cyst	50 mm (diameter)	Single EBUS-TBNA, unknown materials	Yes	NA	No	3 months
Li et al., 2017 [51]	1	Thyroid cyst	11 × 14 mm	Single EBUS-TBNA, 22 Ga	Yes	NA	No	7 weeks

EUS-Guided Drainage (Single Aspiration and Transmural Drainage)

Several factors may influence the feasibility of endoscopic treatment in the mediastinal region. The tubular shape of the esophagus and the elastic property of the GI wall combined allow the endoscope tip to get closer to a remote anatomical region. Additionally, therapeutic scopes are provided with wider working channels so that a broader spectrum of devices is enabled, thereby allowing several alternative strategies to be considered. Mediastinal collections treated by EUS guidance are located in the posterior or middle (or paraesophageal) mediastinum. The esophagus is located at the posterior pole of the middle mediastinum, so access is confined to anterior paraesophageal structures corresponding to the middle mediastinum and posterior mediastinum region. The anterior mediastinum is limited by vascular structures and trachea and is far away from the esophagus, so these regions are best approached by other techniques. Several variables such as lesion features (size and solid component) and the clinical scenario (fever and sepsis) are taken into consideration when deciding upon the best strategy via EUS.

EUS-Guided Single Aspiration

EUS-guided single aspiration is defined as a needle puncture followed by total or near-total content aspiration to collapse the lesion volume; these steps allow for shortening the procedure duration in a safe way. This simple interventional approach is documented in several reports showing acceptable efficacy, reducing the risk of procedure complications compared to transmural drainage. However, the likelihood of recurrence on follow-up is real [18-21].

Table 6 shows a case series involving this endoscopic approach to mediastinal lesions [18,19].

**Table 6:** Mediastinal lesions treated using EUS-guided single aspiration * Disappearance of the lesion or enough reduction to exclude further procedures † Three patients were reported in the original article; in the third patient (not included in our list), only diagnostic EUS-FNA of a mediastinal lesion suggestive of malignancy was performed EUS-FNA: endoscopic ultrasound-guided fine needle aspiration; NA: not applicable

Author, year	Number of patient(s)	Diagnosis	Size	Materials	Clinical success*	Stent removal	Adverse events	Follow-up
Fritscher-Ravens et al., 2000 [18]	2†	Mediastinal abscess	25 mm (diameter)	Single EUS-FNA, 22 Ga	Yes	NA	No	6 months
		Traumatic paratracheal hematoma	57 mm (diameter)	Single EUS-FNA, unknown materials	Yes	NA	NA	8 months
Davarashvili et al., 2017 [19]	1	Infected bronchogenic cyst	50 mm (diameter)	Single EUS-FNA, unknown materials	Yes	NA	NA	4 years

Although EUS-guided single aspiration represents a lower part of the series, it may offer benefits such as endoscopic drainage and obtaining a sample for culture at the same time. The strengths of this option are that it does not need great technical skill and it is less risky than transmural drainage. Moreover, if clinical outcomes are favorable, no further endoscopy is needed.

EUS-Guided Transmural Drainage

This approach is based on temporary stent (plastic or metal) placement to maintain proper drainage between the two cavities, the lesion, and the digestive lumen. EUS-guided transmural drainage of a mediastinal collection is the most common endoscopic treatment approach since its efficacy is well recognized in other body regions as well, such as the abdomen. Table 7 summarizes mediastinal lesions treated by EUS-guided transmural endoscopic drainage [22,23,44-47].

**Table 7:** Mediastinal lesions treated using EUS-guided transmural endoscopic drainage * Disappearance of the lesion or enough reduction to exclude further procedures ‡ Total number of patients treated by each technique LAMS: lumen-apposing metal stent; NA: not applicable

Author, year	Number of patient(s)	Diagnosis	Size	Materials	Clinical success*	Stent removal	Adverse events	Follow-up
Kahaleh et al., 2004 [44]	1	Postoperative mediastinal abscess	40 × 28 mm	Single pigtail 7 Fr	Yes	3 months	NA	3 months
Jonas et al., 2005 [45]	1	Metastatic cyst	30 mm (diameter)	Double pigtail 7 Fr	Yes	NA	NA	18 months
Wehrmann et al., 2005 [22]	20	Paraesophageal abscess formation	>20 mm	Double pigtail 8,5 Fr (4)‡	Yes, 100% (04/04)	NA	NA	12 months (3-40)
				Necrosectomy (Dormia, lavage) (15)‡	Yes, 100% (15/15)	NA	1 death, 6%	
				Abscess access failed (1)‡	No	NA	NA	
Saxena et al., 2014 [46]	1	Postoperative mediastinal abscess	63 × 46 mm	Two double pigtail 7 Fr	Yes	4 weeks	No	3 months
Kawaguchi et al., 2014 [47]	1	Infected bronchogenic cyst	90 × 70 mm	Nasocystic catheter 6 Fr	Yes	3 days	NA	5 months
Consiglieri et al., 2015 [23]	1	Postoperative mediastinal abscess	60 × 50 mm	LAMS 10 × 10 mm	Yes	7 days	NA	2 years

It has shown good results in abdominal lesions; in addition, it is considered the treatment of choice for abdominal lesion drainage in peripancreatic collections. This strategy directed to mediastinal lesions appeals to the abovementioned experience (EUS-guided transesophageal drainage), and the outcomes are acceptable. It seems that the presence of an abscess, likely in the form of a heterogeneous and solid-content lesion, induced the need to perform transmural drainage and, eventually, the creation of a stoma. Although these are case reports, a highlight is a German study that included 15 patients with paraesophageal abscesses treated by EUS-guided transmural technique, with the creation of esophagostomy and routine endoscopic washing, and achieving clinical success in all the patients included [22].

Given the low-level experience reported, it is difficult to recommend a specific stent; however, the majority of cases described involved plastic single- or double-pigtail stent use, likely to avoid local complications related to the use of self-expandable metal stents [23].

Regarding the safety of EUS-guided transmural drainage, two adverse events were reported. One was pneumothorax while performing transesophageal puncture, which was controlled during the procedure [24] The other was a pulmonary artery embolism unrelated to the technique the day after the procedure, with a fatal outcome [22].

EUS-guided transmural drainage is not only effective for lesions originating in the mediastinum; there is also evidence of mediastinum-extended abdominal lesions successfully treated using transmural EUS guidance.

Table 8 shows abdominal lesions with mediastinal extension treated using endoscopic approach [20,21,24,52-66].

**Table 8:** Mediastinal extended abdominal collections drained using an endoscopic approach * Disappearance of the lesion or enough reduction to exclude further procedures † Twelve patients in the original article; one patient refused treatment (not included in our list) § Abstract only EUS-CD: endoscopic ultrasound-guided cyst drainage; EUS-FNA: endoscopic ultrasound-guided fine needle aspiration; LAMS: lumen-apposing metal stent, NA: not applicable

Author, year	Number of patient(s)	Diagnosis	Size	Materials	Clinical success*	Stent removal	Adverse events	Follow-up
Mohl et al., 2004 [52]	1	Pancreatic pseudocyst	NA	Double pigtail 7 Fr	Yes	2 weeks	NA	8 months
Komtong et al., 2006 [53]	1	Pancreatic pseudocyst	40 mm	Transpapillary pancreatic stent 10 Fr	Yes	60 days	NA	6 months
Săftoiu et al., 2006 [54]	1	Pancreatic pseudocyst	150 mm	Plastic stent 10 Fr	Yes	30 days	No	3 months
Gupta et al., 2007 [20]	1	Pancreatic pseudocyst	190 × 120 mm	Single EUS-FNA, 19 Ga	Yes	NA	NA	3 months
Trevino et al., 2009 [55]	3	Pancreatic pseudocyst	8 × 6 cm	Nasocystic stent 7 Fr	Yes	4 days	No	3 years
			7 × 6 cm	Double pigtail 7 Fr	Yes	8 weeks	No	2 years
			6 × 5 cm	Double pigtail 7 Fr	Yes	8 weeks	No	1 year
Mallavarapu et al., 2001 [56]	2	Pancreatic pseudocyst	NA	Transpapillary pancreatic stent 7 Fr	Yes	6 weeks	No	20 months
			NA	Transpapillary pancreatic stent 8.5 Fr	Yes	3 weeks	No	2 years
Bhasin et al., 2012 [57]	11†	Pancreatic pseudocyst	20-80 mm (median: 40 mm)	Naso-pancreatic drain 5 Fr (5) ‡	Yes, 100% (5/5)	8 weeks	No	4 months to 10 years
				Pancreatic stent 5 Fr (5) ‡	Yes, 100% (5/5)	8 weeks	No	
				Pancreatic sphincterotomy (1) ‡	Yes	NA	No	
Gornals et al., 2012 [24]	1	Pancreatic pseudocyst	80 × 50 mm	LAMS 10 × 10 mm	Yes	7 days	Pneumothorax	6 months
Sugimoto et al., 2014 [58]	1	Pancreatic pseudocyst	NA	Double pigtail 7 Fr plus naso tube 5 Fr	Yes	NA	No	10 days
Mishra et al., 2016 [59]	1	Pancreatic pseudocyst	56 × 36 mm	NA	NA	NA	NA	NA
Nasa et al., 2016 [21]	1	Pancreatic pseudocyst	NA	Single EUS-FNA, 19 Ga	Yes	NA	NA	6 months
Dabkowski et al., 2017 [61]	1	Pancreatic pseudocyst	NA	Pancreatic stent	Yes	NA	No	12 months
Takayanagi et al., 2018§ [60]	1	Pancreatic pseudocyst	NA	Transgastric EUS-CD	Yes	NA	No	NA
Pizzicannella et al., 2019 [62]	1	Necrotic pancreatic fluid collection	NA	LAMS	Yes	NA	No	NA
Nakamura et al., 2021 [63]	1	Pancreatic pseudocyst	NA	EUS-guided drainage and naso-pancreatic stent	Yes	NA	No	NA
Aritake et al., 2021 [64]	1	Pancreatic pseudocyst	NA	EUS-guided drainage, stent	Yes	NA	Bronchial fistula and esophageal stricture	4 months
Inomata et al., 2022 [65]	1	Pancreatic pseudocyst	NA	Naso-pancreatic drainage	Yes	NA	No	NA
Watanabe et al., 2022 [66]	1	Pancreatic pseudocyst	NA	Naso-pancreatic drainage (5 Fr)	Yes	NA	Recurrence at 6 months	27 months

The main etiology of the lesions was pancreatic pseudocyst, which, as noted above, constitutes a minor but feasible extension. The endoscopic strategy is diverse, with the EUS-guided drainage being the most common approach. Other strategies include transpapillary pancreatic treatment and EUS-guided single aspiration. Clinical outcomes are promising for such an uncommon condition, as is the safety profile. Pneumothorax was reported while placing a lumen-apposing stent that was successfully treated by intercostal drainage. In spite of this, the thoracic surgeon who performed the procedure thought this was a complication from orotracheal positive pressure [24,54,61].

**Figure 2:**
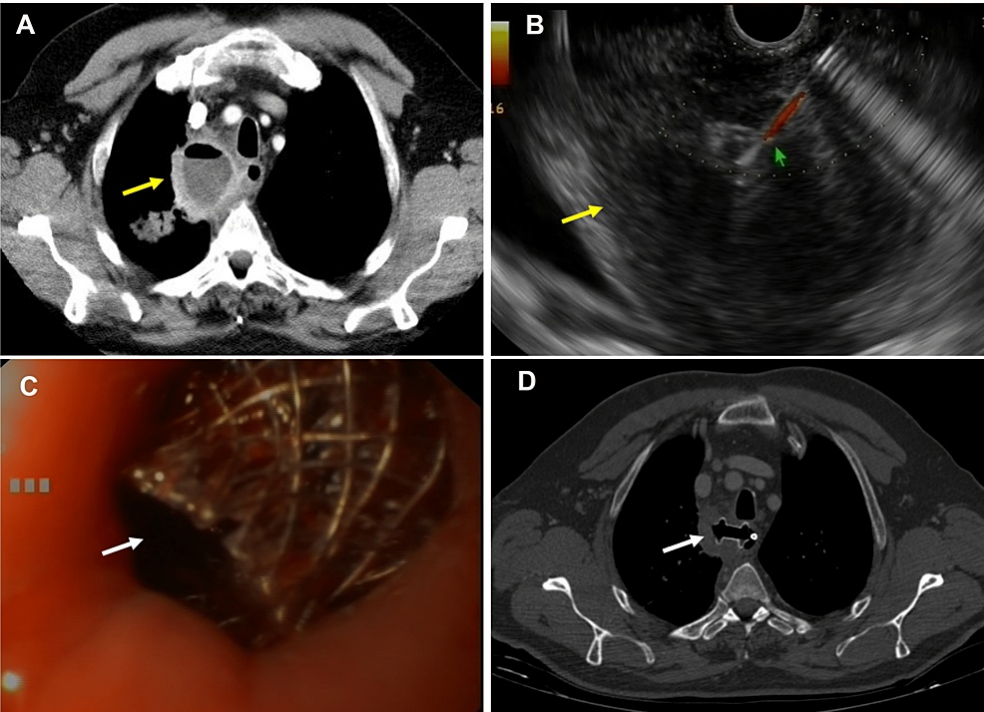
Transmural drainage of mediastinal collection A: Computed tomography scan image of an infected mediastinal collection (note the air-fluid level, yellow arrow). B: Image of endosonography-guided transmural drainage of a mediastinal abscess (yellow arrow); EUS-guided aspiration using a 19 G needle (green arrow) was performed to obtain a sample. C: Endoscopic view of the transesophageal drainage of a mediastinal lesion by placing a lumen-apposing stent (white arrow). D: Computed tomography image of a lumen-apposing stent placed for mediastinal collection of transesophageal drainage (white arrow).

Surgical Approach

Mediastinal surgery constitutes the treatment of abscesses within the mediastinum with the highest success rate. Moreover, since the 1990s, the surgical approach has had new technologies based on video images available, allowing less invasive access compared to open surgery, with a similar recurrence rate [29]. Although these procedures are now less aggressive than conventional approaches, they carry a surgical and anesthesia-related morbidity risk.

## Conclusions

The management of mediastinal collections in which a drainage is required includes a surgical approach and minimally invasive nonsurgical procedures such as percutaneously guided imaging and interventional endoscopy. The nature and location of the collection will condition the selection of the strategy.

Furthermore, although scientific evidence of EUS-guided therapy is limited, it is a promising, minimally invasive technique. The most common mediastinal collections treated by EUS are abscesses and pseudocyst (migrated to the mediastinum) located in the posterior compartment. Basically, EUS-guided drainage of mediastinal collections includes two techniques: collapse by single aspiration (no stent) and transmural drainage (with stent deployment).

Single aspiration can be used by EBUS or EUS, depending on the collection site and accessibility. This is a well-acceptable, non-risky technique, but the potential relapse rate may be higher.

A EUS-guided transmural (transesophageal) drainage differs because of the necessity of stenting, using plastic stents or LAMS. Despite it being more aggressive and associated with complications, the outcomes in long term are superior.

Decisions on therapy strategy should be made by a multidisciplinary committee including surgeons, radiologists, and endoscopists.
